# *Trypanosoma cruzi* Infection in Genetically Selected Mouse Lines: Genetic Linkage with Quantitative Trait Locus Controlling Antibody Response

**DOI:** 10.1155/2014/952857

**Published:** 2014-08-13

**Authors:** Francisca Vorraro, Wafa H. K. Cabrera, Orlando G. Ribeiro, José Ricardo Jensen, Marcelo De Franco, Olga M. Ibañez, Nancy Starobinas

**Affiliations:** Laboratório de Imunogenética, Instituto Butantan, Avenida Vital Brasil 1500, 05503-900 São Paulo, SP, Brazil

## Abstract

*Trypanosoma cruzi* infection was studied in mouse lines selected for maximal (AIRmax) or minimal (AIRmin) acute inflammatory reaction and for high (H_III_) or low (L_III_) antibody (Ab) responses to complex antigens. Resistance was associated with gender (females) and strain—the high responder lines AIRmax and H_III_ were resistant. The higher resistance of H_III_ as compared to L_III_ mice extended to higher infective doses and was correlated with enhanced production of IFN-*γ* and nitric oxide production by peritoneal and lymph node cells, in H_III_ males and females. We also analyzed the involvement of previously mapped Ab and *T. cruzi* response QTL with the survival of Selection III mice to *T. cruzi* infections in a segregating backcross [F1(H_III_×L_III_) ×L_III_] population. An Ab production QTL marker mapping to mouse chromosome 1 (34.8 cM) significantly cosegregated with survival after acute *T. cruzi* infections, indicating that this region also harbors genes whose alleles modulate resistance to acute *T. cruzi* infection.

## 1. Introduction

The protozoan* Trypanosoma cruzi*, the causative agent of Chagas' disease in humans, parasitizes several other mammalian species. After infection with* T. cruzi*, the parasites survive and multiply in nucleated cells as amastigotes, eventually reaching the bloodstream as trypomastigote forms. The acute infection phase is characterized by high levels of circulating parasites, while parasite proliferation is contained during the chronic phase [1].

Innate immune responses play critical roles in the control of parasite spreading and host survival. Toll-like receptor (TLR) family of pattern recognition receptors (PRRs) plays a central role in the recognition of* T. cruzi* by the immune system [2]; TLR4 [3], TLR2 [4–6], TLR9 [7], and TLR7 [8] initiate a signaling cascade that culminates in the activation of proinflammatory genes which are important for resistance to* T. cruzi* infection [9].

NOD1, a member of the cytosolic NOD-like receptor (NLR) family, also plays a role in controlling* T. cruzi* infection;* Nod1*−/− mice were shown to be very susceptible to* T. cruzi,* succumbing to the infection and displaying higher parasitemia and parasite loads in the spleen and heart tissues [10].

Recent works suggest that ASC inflammasomes are critical determinants of host resistance to* T. cruzi* infection [11]; moreover NLRP3 inflammasome controls parasitemia by inducing NO production via a caspase-1-dependent, IL-1R-independent pathway [12].

The early control of replication depends largely on nitric oxide (NO) induction in macrophages mediated by gamma-interferon (IFN-*γ*) and tumor necrosis factor alpha (TNF-*α*). IFN-*γ* is synthesized shortly after infection, mainly by IL-12 and TNF-*α* activated NK cells [13–16]. The* in vivo* inhibition of iNOS results in increased susceptibility to parasites [17, 18].

The effector mechanisms that control parasite loads and survival during the acute infection phase also depend on specific cell-mediated immune responses. Mice depleted or deficient in CD4 or CD8 lymphocytes show early mortality and increased numbers of parasites in their bloodstreams and tissues [19–21]. As a mechanism of evasion during* T. cruzi* replication they release immunomodulatory molecules that delay parasite-specific responses mediated by effector T cells [22].

Resistance to* T. cruzi* infection in humans as well as mice may vary according to the genetic background of the host and the virulence of the parasite strain [23, 24]. Genetic control of responses to* T. cruzi* is governed by multiple genes, and mice of different strains can develop infections that evolve towards either early death or a chronic phase [25].

Silva et al. [26] recently analyzed the susceptibility of several inbred mouse lines to infection with the Y strain of* T. cruzi* and found that susceptibility varied among those lines, especially between A/J and C57BL/6 mice. A/J mice are extremely susceptible, with 100% death rates, whereas C57BL/6 mice are resistant. Data obtained with an F1 (A/J x C57BL/6J) population suggested the existence of one or more loci mapping on chromosome X that contribute to resistance to* T. cruzi* infections.

In addition to these inbred mouse lines, the involvement of different genetic backgrounds in infection control has been analyzed in mice lines selected for either high (H) or low (L) antibody responses and maximal (AIRmax) and minimal (AIRmin) acute inflammatory reactivity (AIR). Starting from a genetically heterogeneous founder population (F_0_) of Albino Swiss mice, the selection of lines for antibody responsiveness (named Selection III) was carried out using assortative mating in successive generations based on secondary antibody response to* Salmonellae* flagellar antigens. This bidirectional selective pressure resulted in the accumulation of alleles at multiple quantitative trait loci (QTLs) in each H and L line endowed with opposite modulatory effects on the various steps of antibody biosynthesis [27]. The differences in antibody responses between H_III_ and L_III_ lines are not restricted just to the selection immunogen but encompass a wide range of complex antigens, showing evidence for multispecific effects of the relevant genes [28, 29]. Genetic analyses indicated that 5–10 QTLs regulate the antibody production phenotype, and a QTL mapping experiment using microsatellite markers yielded three highly significant QTLs on chromosomes 3, 8, and 9 [30]. H_III_ and L_III_ lines also show extreme divergence in other phenotypes, such as skin carcinogenesis [31] and pristane induced arthritis—PIA. Our group successfully detected a PIA-susceptibility QTL on chromosome 3 by examining the cosegregation of the most significant Ab QTL markers with arthritis phenotypes [32].

Selection for acute inflammatory response was carried out in a similar manner, using polyacrylamide beads (Biogel P-100), with induced local inflammatory influx and exudated protein concentrations as the selection phenotypes [33, 34]. Analysis of this selective process indicated that AIR regulation involves at least 11 QTLs [35]. Significant interline differences were also observed in response to several phlogistic agents, including carrageenan, zymosan, and inactivated bacteria [35]. These mouse lines have been used to study the effects of genetic control of nonspecific immunity on susceptibility to neoplastic [36], autoimmune [37], and infectious diseases [38].

The selective process did not affect specific immune responses, as both AIRmax and AIRmin mice produced similar amounts of antibodies after immunization with optimal doses of complex antigens (such as heterologous proteins and bacterial antigens). Cell-mediated immune responses, such as T-cell specific proliferation and delayed-type hypersensitivity reactions, were also similar in both lines. On the other hand, these lines differ in natural resistance to pristane induced arthritis [37], various bacterial infections [38], lung [39], kidney [40], and colon [41] chemical carcinogenesis, as well as wound-healing capacities [42].

The relative contributions of innate and specific immune responses to* T. cruzi* infections have not yet been determined, nor their genetic influences on infection susceptibility. In this study we analyzed the relationships between the genetic controls involved in antibody production and inflammatory responses and resistance to* T. cruzi* infection by examining the course of parasite infection in mice selected for high (H_III_) and low (L_III_) antibody responses (Selection III) or for maximal (AIRmax) and minimal (AIRmin) acute inflammatory responses (AIR).

## 2. Materials and Methods

### 2.1. Mice and Crosses

Male and female 8–12-week-old mice were used in all experiments. All stock mice and crosses used were developed and maintained at the animal facilities of the Immunogenetics Laboratory at the Butantan Institute, São Paulo State, Brazil. All animals received humane care according to criteria outlined in the Ethical Principles in Animal Research Guidelines adopted by the Brazilian College of Animal Experimentation. Experiments were approved (protocol no. 066/02) by the Committee on Ethics in the use of Animals of the Biomedical Sciences Institute—USP.

AIRmax and AIRmin mice were selected from a polymorphic foundation population constructed by the balanced intercrossing of eight inbred mouse lines (A/J, DBA2/J, SWR/J, CBA/J, SJL/J, BALB/cJ, P/J, and C57BL/6J), as described in detail elsewhere [35]. Although the formal stock designations are Ibut : AIRH and Ibut : AIRL, we refer to them in this paper and in previous publications as AIRmax and AIRmin, respectively.

The selection for high (H_III_) and low (L_III_) antibody responder mice (Selection III) was described in detail elsewhere [27].

Inbred H_III_ and L_III_ lines derived from their respective outbred stocks were used to produce F1  (H_III_ × L_III_) hybrid mice and 242 backcrossed (F1 × L_III_) segregating (Bc-L) mice for genetic studies.

Nonselected Swiss Albino male mice purchased from the Central Animal Facilities at the Butantan Institute were used for* in vivo* maintenance of* T. cruzi*.

### 2.2. Experimental Infections, Mortality Rates, and Parasitemia Determinations

The CL strain of* Trypanosoma cruzi* was used in all experiments. The parasite was maintained* in vivo* by serial passage of the parasite blood forms in Swiss Albino mice.

Mice were s.c. infected with* T. cruzi* blood forms diluted at the indicated concentrations in 100 *μ*L PBS buffer and monitored daily for deaths. Parasitemia levels were determined by hemocytometer counts of trypomastigotes in fresh blood samples diluted in ammonium oxalate (1%).

### 2.3. Cell Cultures

Mice were euthanized at 0, 7, 15, and 20 days after infection with 1 × 10^2^ parasites. Their peritoneal cavities were washed with 5 mL of PBS under sterile conditions, and the cells were pelleted and then resuspended in complete RPMI-1640 medium (RPMI-1640 supplemented with 2 mM L-glutamine, 10 g/mL gentamicin, and 10% fetal calf serum). The cells (2 × 10^5^/well) were cultured for 48 h at 37°C and 5% CO_2_ in a final volume of 100 *μ*L/well in 96-well flat-bottom plates with or without stimulation with ConA (2.5 *μ*g/mL).

Lymph node and spleen cell suspensions were obtained by homogenizing those organs in glass grinders with RPMI-1640 medium under sterile conditions and then washing and diluting the cells to 1 × 10^7^ cells/mL in modified Click medium (RPMI-1640 supplemented with 0.05 mM 2-mercaptoethanol, 2 mM L-glutamine, 1 mM sodium pyruvate, 10 g/mL gentamicin, and 2% heat-inactivated normal mouse serum) to a final volume of 1 mL/well in 24-well flat-bottom plates. The cells were stimulated with ConA (2.5 *μ*g/mL) and cultured for 48 h at 37°C and 5% CO_2_. Cell-free culture supernatants were harvested and stored at −20°C.

### 2.4. IFN-*γ* Detection

Unlabeled XMG1.2 (5 *μ*g/mL) and biotinylated AN-18 (5 *μ*g/mL) rat anti-mouse IFN-*γ* monoclonal antibodies were used in two-site sandwich ELISA assays using an alkaline avidin-phosphatase and p-nitrophenyl phosphate substrate in Tris-NaCl buffer to detect IFN-*γ* contents in supernatants of 48-hour cultured lymph node or spleen cells. Absorbance at 405 nm was measured in a Multiskan MS plate reader (Labsystems, Finland), and IFN-*γ* concentrations were determined by comparisons with a standard curve obtained from serial dilutions of recombinant IFN-*γ*.

### 2.5. Nitric Oxide (NO) Quantification

Forty-eight-hour cell-free peritoneal culture supernatants were assayed for NO_2_ using the Griess reaction. Briefly, 50 *μ*L of culture supernatant was incubated with 50 *μ*L of a mixture of 1% sulfanilamide and 0.1% N-(1-naphthyl) ethylenediamine dihydrochloride in 2.5% orthophosphoric acid (H_3_PO_4_) at room temperature for 10 min. Absorbance was measured at 540 nm using a Multiskan MS plate reader (Labsystems); the micromolar concentrations of NO_2_ were determined by interpolation from a NaNO_2_ standard curve.

### 2.6. Quantification of* T. cruzi* DNA in Cardiac Muscle by Real-Time PCR

Genomic DNA from mouse cardiac muscle was isolated using DNeasy Tissue kit (Qiagen, Germany) following the manufacturer's instructions. The primer pairs TCZ-F: 5′-GCT CTT GCC CAC CMG GGT GC-3′ (where M = A or C) and TCZ-R: 5′-CCA AGC AGC GGA TAG TTC AGG-3′ were used in qPCR to detect the target* T. cruzi* 195-bp repeat DNA in the cardiac muscles of infected mice. In parallel, primers for the murine specific TNF-single copy genomic sequence (TNF-5241: 5′-TCC CTC TCA TCA GTT CTA TGG CCC A-3′ and TNF-5411: 5′-CAG CAA GCA TCT ATG CAC TTA GAC CCC-3′) were used in a parallel assay as an internal control to normalize the amount of host DNA loaded in each reaction, as described by Cummings and Tarleton [43].

Normalization was obtained by calculating the ratios of the concentrations of target* T. cruzi* DNA and murine TNF- reference gene DNA in the same tissue sample.

Real-time PCR was carried out with 65 ng of sample DNA, 12.5 *μ*L QuantiTect SYBR Green PCR Master Mix (Qiagen), 0.5 M of each primer, and nuclease-free water to a final volume of 25 *μ*L. The reactions were run in a PTC-200 thermocycler (MJ Research, Inc., USA) with an initial step of 15 min at 95°C followed by 50 denaturation cycles (20 s at 95°C), annealing (20 s at 55°C), and extension (1 min. at 72°C). Fluorescence intensity was detected at the end of each extension phase using a Chromo 4 detector (MJ Research). Melt curves of the products were obtained after the amplification phase. All data was analyzed using Opticon Monitor Analysis Software v2.03 (MJ Research).

In order to estimate target* T. cruzi* DNA concentrations (and therefore the parasite load of each sample), a standard curve was constructed through serial dilutions (ranging from 10,000 to 10 parasite equivalents) of gDNA obtained from uninfected cardiac tissue with 10^4^ *T. cruzi* trypomastigotes artificially added. The standard curve derived from these dilutions (log transformed) indicated the amounts of parasite equivalents in each sample (adapted from [43]).

### 2.7. Genotyping the Polymorphic Microsatellite Markers of Antibody-Controlling QTL (Ab QTL) in Selection III

For genomic DNA extraction, frozen mouse tail tips were incubated at 65°C for 1 h in 100 *μ*L of lysis buffer containing 50 mM Tris-HCl (pH 8.0), 10 mM EDTA (pH 8.0), 0.5% SDS, with 1.5 mg/mL proteinase K (Invitrogen). Following another addition of 100 *μ*L of lysis buffer, incubation for 15 min, and centrifugation (13,000 ×g/10 min), the supernatants were mixed with 0.1 volumes of 3 M sodium acetate (pH 5.2) and 2 volumes of 100% ethanol. The precipitated DNA was washed, dried at room temperature, and dissolved in sterile nuclease-free water.

The genotyping of the Ab QTL microsatellite markers, as described by De Souza et al. [30], was carried out by PCR amplification of 100 ng of DNA using specific primers (Research Genetics, Birmingham, CA). PCR reactions were incubated for 2 min at 94°C, followed by 35 cycles of 30 s/94°C, 35 s/55–57°C, and 45 s/72°C, followed by a final extension for 7 min at 72°C. Individual genotypes of the 242 backcrossed (Bc-L_III_) mice for each marker were determined by comparing their PCR fragment sizes (as visualized in 4.5% agarose gels) with those of the parental lines.

### 2.8. Statistical and Genetic Analyses

Groups of infected mice were submitted to Survival Analysis. Means were compared by analysis of variance (ANOVA) and multiple comparison Tukey post hoc tests. Differences between the groups were indicated when *P* < 0.05.

Individual genetic and phenotypic data from the 242 Bc-L_III_ segregant mice were analyzed using MapManager QTX software [44, 45] to determine the significance of the association between marker genotypes and the* T. cruzi* infection phenotypes. Due to the striking gender difference in infection survival, the log⁡_*n*_⁡ normalized data was analyzed considering sex as a covariate for traits.

Critical LRS (likelihood ratio statistic) values corresponding to suggestive (*P* < 0.63), significant (*P* < 0.05), or highly significant (*P* < 0.001) linkage [46] were determined by Random Permutation Testing [47].

## 3. Results

### 3.1. Mortality Rates

**Figure 1:**
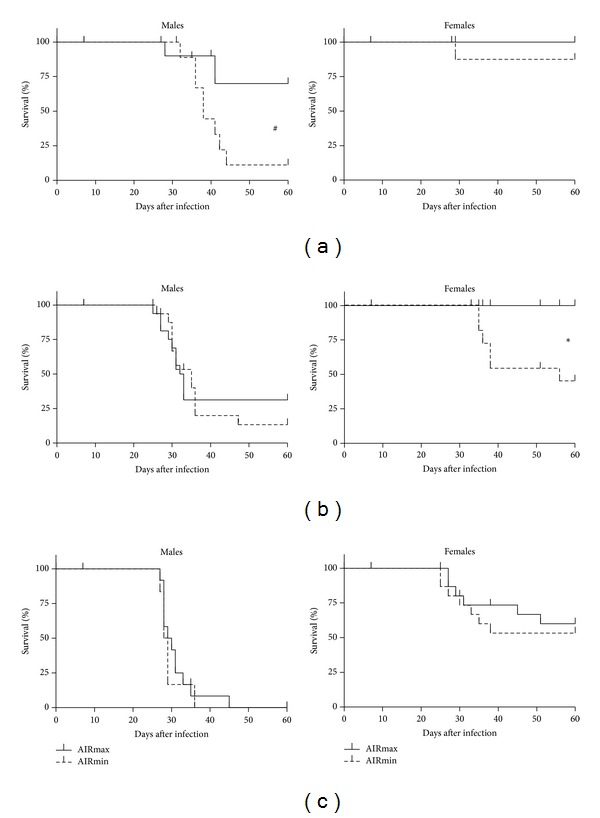
Survival curves of AIR mice after* T. cruzi* infection. AIRmax and AIRmin mice were infected (s.c.) with 10 (a), 10^2^ (b), or 10^3^ (c) of CL strain trypomastigote forms. Interline differences evaluated by survival curve analyses of each group (*n* = 8) are indicated for males (^**#**^ *P* = 0.04) and females (^∗^ *P* = 0.003).

**Figure 2:**
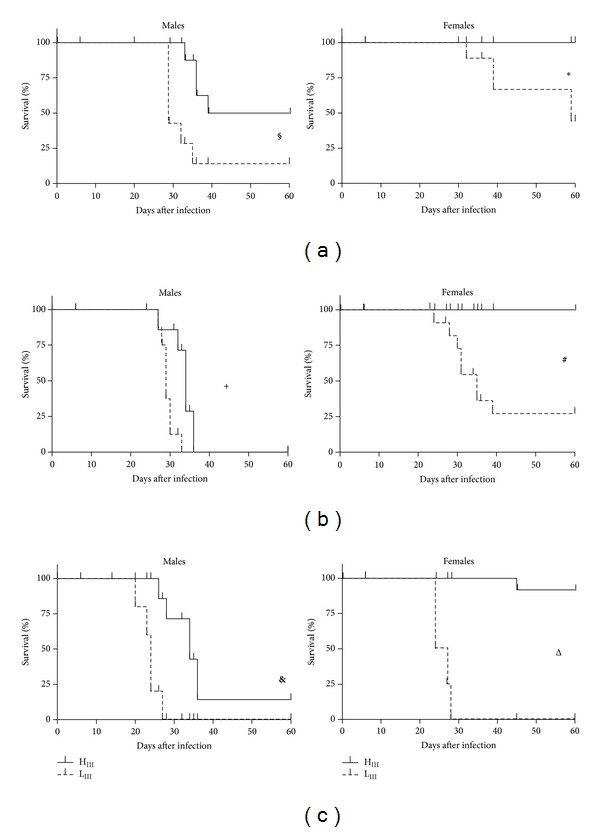
Survival curves of Selection III mice after* T. cruzi* infection. H_III_ and L_III_ mice (outbred stock) were infected (s.c.) with 10 (a), 10^2^ (b), or 10^4^ (c) of CL strain trypomastigote forms. Interline differences evaluated by survival curve analyses of each group (*n* = 8) are indicated for males (^**§**^ *P* = 0.018; ^**+**^ *P* = 0.05; ^**&**^ *P* = 0.008) and females (^∗^ *P* = 0.015; ^**#**^ *P* = 0.0008; ^Δ^ *P* < 0.0001).

Mortality rates, monitored at doses ranging from 10 to 10^4^ blood forms, showed marked gender-related differences in susceptibility to acute infection in all of the mouse lines analyzed. Males were more susceptible than their respective littermate females (Figures 1 and 2). In addition, we observed divergent levels of resistance in both the AIR (Figure 1) and Selection III (Figure 2) lines.

AIRmax males were significantly more resistant to infection with 10 parasites than AIRmin males (Figure 1(a)), while no differences were observed at higher infective doses (Figures 1(b) and 1(c)). AIRmax females were likewise more resistant than AIRmin females, showing no mortality when challenged with 10 and 10^2^ parasites; AIRmin females inoculated with 10^2^ trypomastigotes showed high mortality rates (Figure 1(b)) and different survival curves. Infection with more than 10^2^ parasites, however, resulted in similar susceptibilities in females of both AIR lines (Figure 1(c)).

Both H_III_ and L_III_ male mice had high and similar mortality rates (Figures 2(a), 2(b), and 2(c)) when infected. L_III_ male mice died earlier than H_III_ animals, however, resulting in significantly different survival curves when infected with 10, 10^2^, and 10^4^ parasites.

Female H_III_ mice were more resistant than L_III_ females independent of the challenge dose, with clearly different survival curves and mortality rates (Figures 2(a), 2(b), and 2(c)). While only one H_III_ female died when challenged with the highest dose, mortality was already significant at the lowest dose among L_III_ females, reaching 100% after challenges with 10^4^ parasites. Female L_III_ mice mortality was similar to that of the extremely susceptible L_III_ males (Figure 2(c)).

### 3.2. Parasitemia

**Figure 3:**
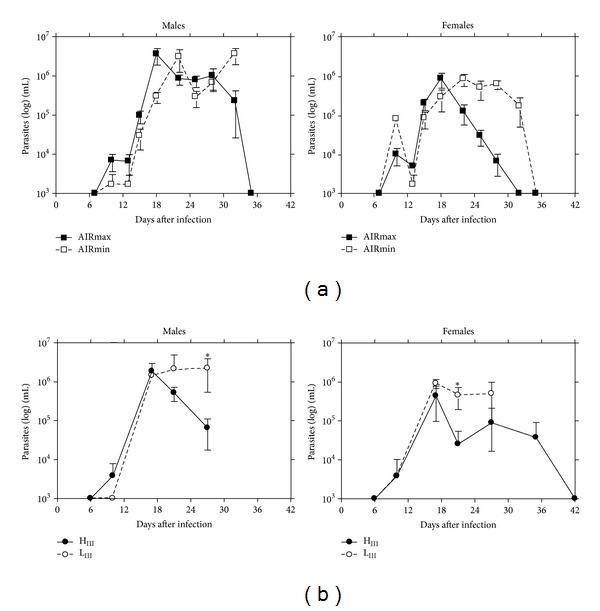
Parasitemia of (a) AIRmax, AIRmin mice (*n* = 6 animals/group), and (b) H_III_, L_III_ mice (*n* = 4 animals/group) during the acute phase of infection with 10^2^ trypomastigote forms of* T. cruzi* (CL strain). The results are expressed as the mean ± SE of each group of males and females and significant differences between AIRmax versus AIRmin and between H_III_ versus L_III_ mouse lines in parasitemia levels are indicated: ^∗^ *P* < 0.05.

Males and females of all mouse lines tested developed similar parasitemia peaks in acute* T. cruzi* infections, independent of initial parasite challenges (representative plots for the 10^2^ dose are shown in Figure 3).

Differences related to both gender and lines were observed in parasite-clearance in these mice. Thus, except for a few AIRmax males that cleared blood parasite forms to undetectable levels, the other males died after acute infection (showing high levels of circulating parasites).

High percentages of females from AIRmin (60%) and L_III_ (100%) susceptible lines died without showing any effective control of parasitemia levels, whereas all females of resistant AIRmax and H_III_ lines cleared blood parasites during acute* T. cruzi* infection.

**Figure 4:**
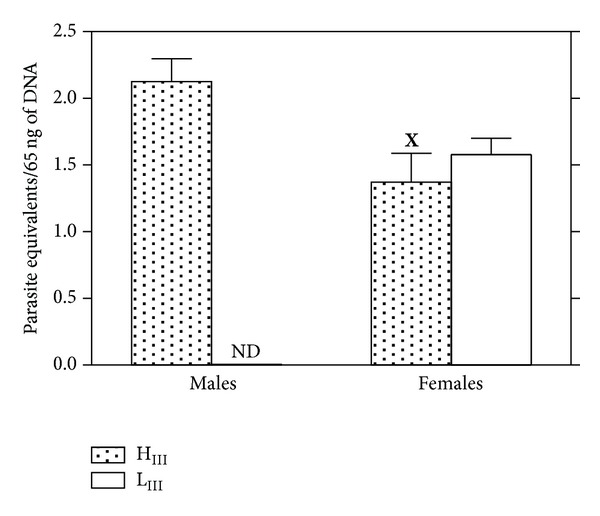
Parasite loads in the cardiac muscles of H_III_ and L_III_ male and female mice 150 days after infection (sc) with 10^3^ trypomastigote forms of the CL strain of* T. cruzi*. Significant differences of the parasite loads in 65 ng of DNA isolated from cardiac tissue are indicated: ^**X**^ *P* = 0.0125 (between H_III_ males and H_III_ females); ND: not determined (all male L_III_ mice die in the acute phase).

Differences in parasitemia control among H_III_ and L_III_ females in the acute infection phase were not correlated with parasite loads in cardiac tissue in the late phase (150 days after challenging) (Figure 4). This correlation could not be determined in males because all L_III_ males died during the acute phase. Gender-related differences were observed in H_III_ mice in terms of the amounts of* T. cruzi* genomic DNA found in their cardiac tissue, with higher values being found in males than in females of both lines (Figure 4). This data indicates that males differ from females not only in terms of the lethality of infection and of parasitemia control, but also in terms of parasite burdens in heart tissue.

### 3.3. Gamma Interferon and NO Production

Production of IFN-*γ* and nitric oxide (NO) in the acute phase of* T. cruzi* infection was investigated to evaluate whether these mediators were associated with the different levels of infection resistance in mice selected for AIR or Ab response phenotypes.

Increases in both IFN-*γ* and NO production were observed in infected AIRmax and AIRmin mice, but the increases were similar in both lines and did not correlate with either strain-related or gender-related differences in resistance (data not shown).

**Figure 5:**
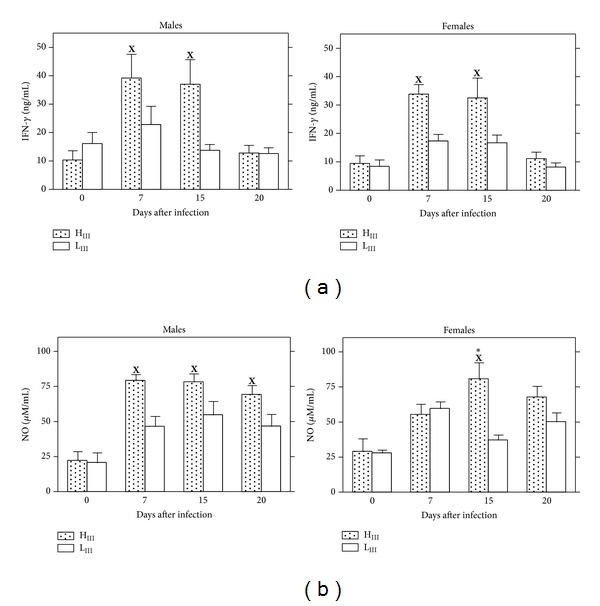
IFN-*γ* (a) and nitric oxide (NO) (b) production by lymph node (a) and peritoneal cells (b) during the acute phase of infection with 10^2^ *T. cruzi* parasites (CL strain) in outbred H_III_ and L_III_ mice. Cells were stimulated in culture with ConA (2.5 *μ*g/mL) for 48 h. Results are expressed as the means ± SE of each group (*n* = 6). Significant differences between infected and control mice (**X**) or between lines (∗) evaluated by ANOVA followed by Tukey multiple comparison tests are indicated when *P* < 0.05.

Lines selected for Ab response, on the other hand, showed distinct IFN-*γ* production patterns that increased significantly at 7 days and remained elevated 15 days after infection in H_III_ males and females; in L_III_ mice, IFN-*γ* production was similar to noninfected control levels at all time points (Figure 5(a)). Our data also suggests that IFN-*γ* production influences NO synthesis during acute infection as increases in this trypanocidal mediator were observed in both H_III_ males and females (Figure 5(b)) after, or at the same time as, IFN-*γ* detection. NO levels did not differ between infected and control L_III_ mice. Similar profiles of IFN-*γ* and NO secretion were observed with lymph node, peritoneal, and spleen cells (data not shown). Interestingly, we observed the same patterns of IFN-*γ* and NO production with males and their littermate females, so that no correlations between the production of these mediators and gender-related resistance to infection could be established in any of the mouse lines studied.

### 3.4. QTL Analyses of Responses to* T. cruzi* Infection

The quantitative trait* loci* (QTLs) controlling Ab production in Selection III lines had been previously mapped in genome-wide screening with polymorphic genetic markers (microsatellites) [18]. We therefore investigated the participation of these chromosomal regions in the response to* T. cruzi* infection. Inbred subpopulations of H_III_ and L_III_ lines that showed the same resistance pattern to* T. cruzi* infections as the outbred parental lines described above were used for this purpose.

**Figure 6:**
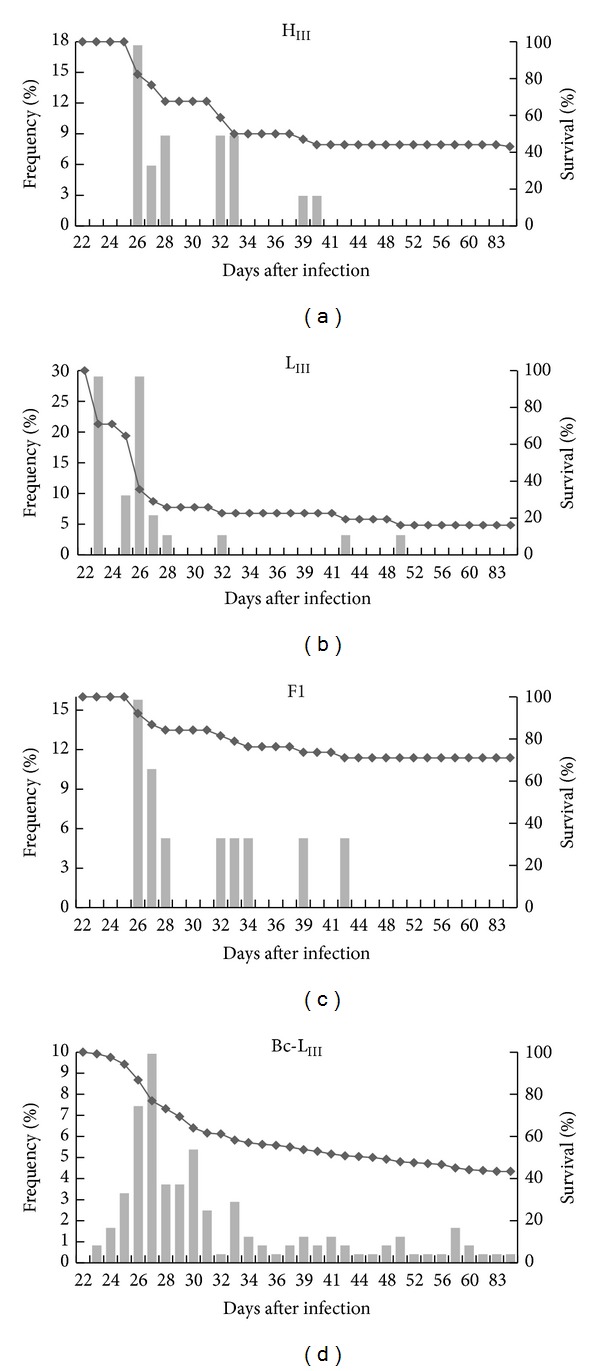
Survival curves and the mortality frequencies each day after* T. cruzi* infection. Males and females of inbred parental H_III_ (a) and L_III_ (b) lines, F1  (H_III_ × L_III_) hybrids (c), and backcrossed Bc-L (F1 × L_III_) mice populations (d). Mice were infected (s.c.) with 10^3^ blood forms of the CL strain of* T. cruzi* and monitored daily for mortality.

F1  (H_III_ × L_III_) hybrids, obtained by reciprocal crosses (H_III_ males X L_III_ females or H_III_ females x L_III_ males), showed identical resistance patterns to* T. cruzi* challenges (data not shown), indicating that there were no maternal or parental effects in determining resistance/susceptibility to infection. On the other hand, there was an overdominance effect of the resistance phenotype, with F1 hybrids showing greater resistance than the parental H_III_ line (Figures 6(a), 6(b), and 6(c)).

Due to the strong overdominance observed in F1 mice infected with 10^2^ parasites (Figure 6(c)) and to a wide range of other challenge doses (data not shown), a segregating population of 242 mice was constructed by backcrossing F1 mice with the susceptible parental L_III_ line (Bc-L). Composite data related to the survival times of males and females from this backcross population (Figure 6(d)) were submitted to genetic analysis, considering sex as a covariate for that trait.

Of all microsatellites tested, the Ab QTL marker located at 34.8 cM on chromosome 1 (*D1Mit303*) attained significant cosegregation values for survival time (Table 1). A more distal peak in chromosome 1 (marked by three adjacent microsatellites) also involved in Ab regulation in H_III_ and L_III_ lines showed a suggestive level of cosegregation with mortality and survival rate.

## 4. Discussion

The present work demonstrated that polygenic regulation leading to high or low antibody production and maximal or minimal acute inflammatory responses affects host resistance against* T. cruzi* infections. Our results showed a positive correlation between the resistance phenotype and mouse lines selected for high responses.

During acute infection, mice from both high-responder lines had lower mortality rates and showed greater capacities to control circulating parasites when compared to low-responder animals. This correlation was more evident among female mice.

Gender differences were also observed in mortality rates, with male mice being more susceptible than females. Male mice have been observed to be more susceptible to acute infections than females in other genetic mouse models, with significantly lower numbers of circulating parasites being observed in the latter [48], and hormones such as estrogen have been observed to reduce mortality in* T. cruzi* infected mice, presumably by their ability to stimulate macrophage activity [49]. Using* Calomys callosus* as an experimental model, Prado Jr. et al. [50, 51] showed that gonadectomy affected the courses of* T. cruzi* infections in females, with high parasitemia levels in the ovariectomized animals as compared to controls and sham-operated groups, indicating that sex hormones can influence natural immune mechanisms. The influence of gender on human susceptibility to* T. cruzi* has similarly been reported [52, 53].

Recent work analyzing an F2 population obtained by intercrossing resistant and susceptible isogenic strains of mice found a significant association between parasitemia and mortality. By analyzing males and females separately, the authors found that males were more susceptible to death but parasitemia was similar in males and females. In fact they obtained a negative correlation of parasitemia with longevity in females but not in males, suggesting that additional factors independent of parasitemia cause early mortality in males during infection with* T. cruzi* [54].

In the present study, the selected mouse lines behaved like other mouse lines with susceptible male mice dying with higher numbers of blood trypomastigotes, while in females this association could not be done. The resistant mice enter the chronic phase without detectable parasites in their bloodstreams.

Some studies demonstrated that antibodies are responsible for the survival of susceptible animals in the initial phase of* T. cruzi* infection and for the maintenance of low levels of parasitemia in the chronic phase [55–57]. Despite the important effector role of antibody in the control of* T. cruzi* infection, resistant lines do not necessarily produce higher levels of specific antibody in comparison to susceptible lines [58, 59]. Elevation of specific antibody in the acute phase* T. cruzi* infection showed no correlation with the survival in isogenic mice. In the other hand, resistance correlated with enhance of some parasite-specific antibodies isotypes, particularly of IgG2b, [60–62].

It has been shown that an X-linked mutation (that prevents B1 cell development and specific and nonspecific immunoglobulins production) of Balb. Xid immunodeficient mice influences resistance to infection. Surprisingly,* T. cruzi* infected Xid mice were more resistant than wild-type mice, and the resistance was associated with the absence of IL-10 secreting B1 cells and increased production of IFN-*γ* [63, 64].

Cellular immune responses are considered important components of resistance to* T. cruzi* infection, with IFN-*γ* as a central mediator that activates NO-dependent parasiticidal mechanisms in macrophages [17, 65, 66].

In the present study, the increased levels of IFN-*γ* observed 7 and 15 days after infection in ConA-stimulated cells from H_III_ mice (Figure 5(a)) (as compared to cells from L_III_ mice) apparently trigger the higher NO levels observed in stimulated cells in those animals, suggesting an association between NO production and infection resistance. However, NO and IFN-*γ* were produced in significant levels in both resistant female and susceptible male mice of the H_III_ and AIRmax and AIRmin lines, indicating that IFN-*γ* and NO secretion are not the only parameters that contribute to gender differences in infection resistance and may not always correlate with survival, so that other mechanisms must be involved.

Wrightsman et al. [25] studied the genetic control of responses to* T. cruzi* infection and observed that multiple genes were involved in the control of parasitemia and survival, with female F1 hybrid mice from crosses of several susceptible and resistant mouse lines surviving the infections, indicating dominance of the resistance genes. Our results confirmed and extended these observations, indicating an overdominance of the resistance phenotype in F1 (H_III_ × L_III_) mice, with females being more resistant than males.

This data is in agreement with other genetic studies in which infection resistance was observed to be inherited in a dominant manner, with heterozygosity in crosses between susceptible and resistant inbred lines (or even between two susceptible lines) increasing resistance [24–26, 67]. Overall, these results indicate that, in addition to polygenic regulation, there is complementarity between different loci in determining the infection resistance phenotype.

The overdominance of the resistance phenotype in Selection III mouse lines led us to choose a segregating population Bc-L, created by backcrossing F1 [H_III_ × L_III_] with the susceptible parental L_III_ mice, to carry out the genetic linkage analysis. Backcrosses are more powerful than intercrosses for examining dominant/recessive traits because they eliminate interference from homozygous dominant genotypes in the analyses [68].

Susceptibility to infectious disease is influenced by multiple host genes, most of which are low penetrance QTLs that are difficult to map. So, the strategy that we chose for genetic analysis was to analyze the linkage between the mortality and survival phenotypes after infection of the Bc-L population with markers that were previously described as implicated in antibody production regulation as well as other markers implicated in resistance to* T. cruzi* infection

Microsatellite markers of QTLs for antibody production mapping on chromosomes 1, 3, 5, 6, 9, 11, and 12 were tested in this linkage analysis [30]. We also genotyped polymorphic microsatellites among lines of Selection III mice that mark chromosomal regions related to resistance to acute infection by* T. cruzi* on chromosome 7 and near the H-2 locus on murine chromosome 17 [67, 69]. Two other markers on chromosome 1 were tested in a region associated with resistance to African trypanosomiasis (*Tir3b*) [70].

We detected a significant association between alleles of an Ab QTL on chromosome 1 (marked by microsatellite* D1Mit303* at 34.8 cM) with the survival phenotype after* T. cruzi* acute infection, obtaining a significant level of cosegregation in this region in spite of the strong influences on the phenotype of environmental factors such as sex hormones and limited population sizes.

Iraqi et al. [70] described one QTL (*Tir3a*) associated with African trypanosomiasis infection adjacent to the abovementioned QTL confidence interval. This region overlaps a QTL that controls antibody production in H_III_ and L_III_ mouse lines as well as a putative QTL for acute inflammatory response mapped in AIRmax and AIRmin mice [71]. Several genes map at the QTL interval that could interfere in the various steps of innate or adaptative immune response regulation.

A candidate gene in this region is* Slc11a1* (solute carrier family 11; proton-coupled divalent metal ion transporters, member 1 (formerly known as* Nramp1*)) which is a major gene regulating control of intracellular pathogen infections such as those caused by* Salmonella* Typhimurium,* Leishmania donovani*, and* Mycobacterium bovis* bacillus Calmette-Guerin (BCG) in mice and humans. This gene is also involved in inflammatory autoimmune diseases [72] and seems to interact with other genes to modulate this phenotype [73], as could be the case with* T. cruzi* infection in mice selected for acute inflammatory reaction.

However, this gene has no influence on the differential response of Selection III mice to* T. cruzi* because both lines have the resistance associated allele of this gene [74]. Despite this, H_III_ and L_III_ lines differ in susceptibility to* Salmonella* Typhimurium showing that the region that controls antibody production marked by the D1Mit303 microsatellite harbor genes involved in resistance to this bacterial infection [74] and also to* T. cruzi*, in absence of* Nramp*1 polymorphism. Also in humans a lack of association between* NRAMP1* gene polymorphism and* T. cruzi* infection was described [75].

Other genes located in this region, such as* Casp8*,* Icos* (induced T-cell costimulator),* CD28*, and chemokine receptors* Cxcr1* and* Cxcr2* (IL-8 receptor) could also be involved in this phenotype by regulating activation of the inflammatory and adaptive immune responses.

Genes evolved in apoptosis like* Casp8* may be implicated in the different resistance pattern we observed. Infection with* T. cruzi* triggers apoptosis of T and B lymphocytes, and lymphocyte apoptosis has immunoregulatory implications for host immune responses [22]. Treatment* in vivo* with a caspase inhibitor reduces lymphocyte apoptosis and improves protective immune responses in mice infected with* T. cruzi* [76].

Other studies assessed the involvement of caspase signaling in thymocyte death during* T. cruzi* infection and showed that caspase-8 and caspase-9 mediate thymocyte apoptosis in* Trypanosoma cruzi* acutely infected mice [77].

Another gene described in this region codes for CD28 molecule that mediates costimulatory signals required for T-cell activation. The involvement of CD28 in the modulation of protective immunity against* T. cruzi* was shown by mediating the activation of both CD4+ and CD8+ T cells, the production of IFN-*γ*, and, as a consequence, the production of NO efficient to control parasite growth during the acute phase of the infection. Mice knockout for CD28 presented high parasitemia and mortality [78]. However, when infected with the low virulent Sylvio X10/4 trypanosome strain CD28-KO mice exhibited resistant phenotype, with no parasitemia or mortality [79].

Chagasic patients lack CD28 expression on many of their circulating T lymphocytes [80].

The role of CXC chemokines in proinflammatory phenotype, developed by* T. cruzi* infection, was shown by experiments, in which tissue culture trypomastigotes activate innate sentinel cells via TLR2, releasing CXC chemokines, which in turn evoke neutrophil/CXCR2-dependent extravasation of plasma proteins, including high molecular weight kininogen, in parasite infected tissues [81, 82].

The QTL on chromosomes 3 and 9 that show the highest cosegregation significance with antibody production levels in H_III_ and L_III_ mice [30] were not involved in* T. cruzi* infection control. In previous studies with parasite infections, the response of Selection III lines to infection by the protozoan* Toxoplasma gondii* showed correlation with the potentiality of specific antibody production of their lines [83]. Also in the course of infection by Y strain of* T. cruzi*, minor mortality rates and more efficient control of parasitemia were associated with significant differences in* T. cruzi* specific IgG antibodies [84]. Herein, using CL strain, we quantified antibodies against* T. cruzi* antigen during the acute phase of infection but no differences in specific antibody production could be detected between lines, in spite of an increase of both IgM and IgG levels at 20 days of infection (data not shown), presumably due to a polyclonal activation induced by this parasite [85, 86].

The chromosome 5 Ab QTL located at 85 cM was not associated with the phenotypes analyzed here, although Graefe et al. [67, 87] described a locus on chromosome 5 at 58 cM associated with male mortality in the acute phase of* T. cruzi* Tulahuen strain infection [87]. Iraqi et al. [70] also described a QTL associated with survival of* T. congolense* infections adjacent to this region (at 42–44 cM). One reason for the failure to detect any association may be the large genetic distance between the marker and the published* T. cruzi* resistance QTL on this chromosome. The markers available in this 40–60 cM interval in the microsatellite panel used for mapping Ab QTL were not polymorphic among the H_III_ and L_III_ lines, and additional markers will need to be tested for polymorphism and any associations before definitive conclusions can be drawn.

The chromosome 11 QTL showed the highest significance among the resistance loci (survival) to the Y strain parasite in inbred mice [67]. These and other authors also described a QTL on chromosome 17 close to the H-2 complex as a determinant of responses to challenge with both* T. congolense* (*Tir1*) [70] and* T. cruzi* [66], although this region was not associated with either* T. cruzi* infection control or antibody production phenotypes in H_III_ and L_III_ mouse lines in the present study [30, 88].

Differential gene expression was analyzed in the spleens of infected susceptible C57BL/6 and resistant (C57BL/6 X DBA/2) F1 mice using microarrays, and the results suggested that the differential transcription of certain genes involved in immune responses and inflammatory processes accounted for the differences in susceptibility to the Tulahuen strain of* T. cruzi* [87].

In humans many genetic linkages and association studies have attempted to identify genetic variations that are involved in immunopathogenesis of Chagas disease. However, causal genetic variants underlying susceptibility remain unknown due to complexity of parasite and host. Susceptibility/resistance to Chagas disease involves multiple genetic variants functioning jointly, each with small or moderate effects [89]. Polymorphism in the* ACTC1* gene of humans contributes to the progression to chronic autoimmune Chagas cardiomyopathy [90], and polymorphisms of other genes that affect several immune parameters such as innate immunity, signal transduction, and T-cell/monocyte migration to inflammatory regions play a role in genetic susceptibility to CCC development [91].

Our data suggest that one out of the several quantitative trait loci that regulate antibody production also contributes to the control of* T. cruzi* infection. Multiple genes control the several steps of antibody synthesis or of infection. The modifications in mechanisms that lead to differential immune response of H_III_ and L_III_ selected lines rather than the produced anti-*T. cruzi* antibodies might play a major role in infection outcome.

Overall, the results of this study demonstrated that an immunomodulatory QTL mapping on mouse chromosome 1 significantly cosegregated with the phenotype of survival time to acute* T. cruzi* infection. Therefore, our data indicates that a region controlling Ab production and inflammation on mouse chromosome 1 harbors genetic factors that also determine resistance to acute* T. cruzi* infections. This region had not previously been implicated with this disease, demonstrating the potential of this genetic model for dissecting complex multigenic regulated traits.

## Figures and Tables

**Table 1:** Significance of cosegregation between mortality and survival time traits with microsatellites markers of Ab regulating QTLs (quantitative trait loci) from Selection III or with candidate region markers of *T. cruzi* infection susceptibility.

Cosegregation test				Trait	
Males and females Bc-L				Mortality	Survival time^d^
Chrom.	Marker	Location		LRS^c^	LRS^c^
		(Mb)^a^	(cM)^b^		
**1**	D1Mit411	33220299–33220410	12.6	1.6	1.6
	**D1Mit303**	**62907596–62907723**	**34.8**	**7.4**	**9.1** ^ #^
	D1Mit286	128551097–128551241	67.0	3.4	3.9
	D1Mit102	147249520–147249632	79.0	5.3	5.6
	D1Mit36	169211172–169211344	92.3	3.9	5.6
	D1Mit149	172699229–172699330	94.2	3.9	5.7

3	D3Mit272	33007369–33007465	15.5	3	3.4
	D3Mit100	97081705–97081845	46.0	1.3	1.5

5	D5Mit122	150116320–150116477	85.0	0	0

6	D6Mit123	N/D	29.0	0.1	0.5
	D6Mit128	83471199–83471322	35.0	0.2	0.1
	D6Mit6	85277319–85277417	35.3	0.4	0.3

7	D7Mit176	63211190–63211340	27.0	0.3	0.1

9	D9Mit90	32500455–32500594	9.0	0.8	0.9
	D9Mit248	58362559–58362688	31.0	0	0
	D9Mit207	60573431–60573577	33.0	0.8	0.4

11	D11Mit4	68609257–68609504	37.0	0.2	0.9

12	D12Mit19	N/D	58.0	1.3	1.1

17	D17Mit177	48558951–48559063	24.0	1.8	1.1

^a^Megabases (mouse genome database); ^b^CentiMorgans (mouse genome database); ^c^likelihood ratio statistic; ^d^survival time (days); N/D: not determined.

Critical LRS statistic values established by Random Permutation Test for the trait.

(i) Mortality: significant ≥ 8.8; highly significant ≥ 15.5; suggestive ≥ 2.7.

(ii) Survival time: ^#^significant ≥ 7.9; highly significant ≥ 14.3; suggestive ≥ 2.8.
